# Concerted regulation of retinal pigment epithelium basement membrane and barrier function by angiocrine factors

**DOI:** 10.1038/ncomms15374

**Published:** 2017-05-19

**Authors:** Ignacio Benedicto, Guillermo L. Lehmann, Michael Ginsberg, Daniel J. Nolan, Rohan Bareja, Olivier Elemento, Zelda Salfati, Nazia M. Alam, Glen T. Prusky, Pierre Llanos, Sina Y. Rabbany, Arvydas Maminishkis, Sheldon S. Miller, Shahin Rafii, Enrique Rodriguez-Boulan

**Affiliations:** 1 Department of Ophthalmology, Margaret Dyson Vision Research Institute, Weill Cornell Medicine, 1300 York Avenue, New York, New York 10065, USA; 2 Angiocrine Bioscience, Inc., 11575 Sorrento Valley Road, Suite 217, San Diego, California 92121, USA; 3 Department of Physiology and Biophysics, Weill Cornell Medicine, 1300 York Avenue, New York, New York 10065, USA; 4 Burke Medical Research Institute, 785 Mamaroneck Avenue, White Plains, New York 10605, USA; 5 Bioengineering Program, DeMatteis School of Engineering and Applied Science, Hofstra University, 1000 Fulton Avenue, Hempstead, New York 11549, USA; 6 Ansary Stem Cell Institute, Department of Medicine, Division of Regenerative Medicine, Weill Cornell Medicine, 1300 York Avenue, New York, New York 10065, USA; 7 Section of Epithelial and Retinal Physiology and Disease, National Eye Institute, National Institutes of Health, 31 Center Drive MSC 2510, Bethesda, Maryland 20892-2510, USA

## Abstract

The outer blood-retina barrier is established through the coordinated terminal maturation of the retinal pigment epithelium (RPE), fenestrated choroid endothelial cells (ECs) and Bruch's membrane, a highly organized basement membrane that lies between both cell types. Here we study the contribution of choroid ECs to this process by comparing their gene expression profile before (P5) and after (P30) the critical postnatal period when mice acquire mature visual function. Transcriptome analyses show that expression of extracellular matrix-related genes changes dramatically over this period. Co-culture experiments support the existence of a novel regulatory pathway: ECs secrete factors that remodel RPE basement membrane, and integrin receptors sense these changes triggering Rho GTPase signals that modulate RPE tight junctions and enhance RPE barrier function. We anticipate our results will spawn a search for additional roles of choroid ECs in RPE physiology and disease.

## Abstract (web summary)

Establishment of the outer blood-retina barrier is a hallmark of retinal development but the contribution of choroid endothelial cells (ECs) is not known. Here the authors show in the developing mouse retina that ECs remodel the basement membrane and lead to enhanced barrier function of retinal epithelial cells.

The retinal pigment epithelium (RPE) is a support cell layer that lies immediately adjacent to the photoreceptors (PRs), the light sensitive cells in the retina (Fig. 1a). The physiology of RPE and PRs is intimately interconnected and vision depends on the proper communication between both cell types. Key roles of RPE include recycling of components of the visual cycle, clearance of PR membrane fragments generated during daily PR renewal, transport of nutrients from the circulation to the subretinal space and evacuation of retinal waste products in the opposite direction^1^. The RPE sits on top of Bruch's membrane (BM), a highly organized-, elastin- and collagen-rich basement membrane that separates RPE from fenestrated choroidal capillaries^2^. A hallmark of retinal development is the establishment of the outer blood-retina barrier (oBRB) by coordinated terminal maturation of RPE, BM and choroid blood vessels. In humans this process takes place *in utero* whereas in rodents it occurs after birth^3^. The oBRB regulates the exchange of nutrients, fluid and waste between the neural retina and the choroid circulation, which is essential for light transduction in PRs (ref. 1). One of the key events during establishment of the oBRB is the acquisition of fully mature RPE tight junctions (TJs), which limit paracellular movement of ions and water across the RPE monolayer and maintain the correct apico-basal distribution of RPE transporters^3^. Both features are key for the correct formation of gradients that drive directional fluid transport from the neural retina to the choroid, essential for the maintenance of retinal homeostasis^4^.

A regulatory role of choroid endothelial cells (ECs) in the establishment of the oBRB is conceivable, given recent studies showing that ECs, beyond their role as blood conduits, constitute instructive niches for parenchymal cell differentiation, regeneration and function^5^. Importantly, there is strong evidence for a role of ECs in the development of structural and functional features by different epithelial cells, such as the acquisition of apico-basal polarization in hepatocytes^6^, the regulation of insulin secretion by pancreatic cells^7^ and the development of proper foot processes by podocytes^8^. Remarkably, mutant zebrafish embryos with disrupted vascular systems, which oxygenate their tissues by diffusion^9^, display defective retinal development^10^. These diverse lines of strong circumstantial evidence led us to test the hypothesis that choroid ECs regulate terminal maturation of the oBRB. Our results describe for the first time a mechanism of communication between choroid ECs and RPE through which EC-secreted factors remodel the RPE basement membrane, which results in modulation of RPE TJs and enhancement of RPE barrier function.

## Results

### Transcriptome of developing and adult choroid ECs

**Figure 1:**
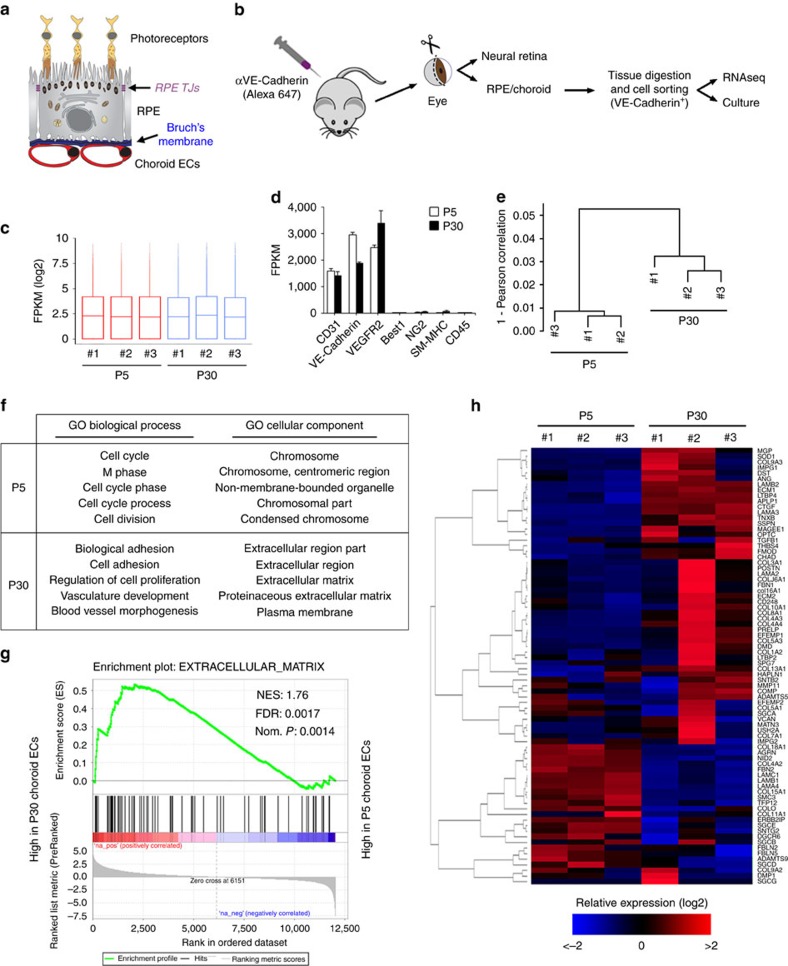
Choroid ECs become enriched in ECM-related transcripts after terminal differentiation. (**a**) Scheme of the outer retina. (**b**) Scheme of choroid EC isolation after intravital EC labelling. Seven animals were used per isolation, and RNAseq was carried out from three independent isolations. (**c**) Boxplots showing gene expression levels (log2 FPKM) of P5 and P30 choroid ECs. (**d**) Analysis of the FPKM (mean+s.d.) corresponding to endothelial (CD31, VE-Cadherin and VEGFR2) and non-endothelial (Best1, RPE; NG2, pericytes; SM-MHC, smooth muscle cells; CD45, haematopoietic cells) markers in P5 and P30 choroid ECs indicated very low levels of contaminating non-ECs in the preparation (*n*=3). (**e**) Hierarchical clustering of FPKM profiles showed separate clustering of P5 and P30 choroid ECs (*n*=3). (**f**) Biological process and cellular component gene ontology (GO) analyses using DAVID software. The five most significant gene sets per condition are shown. P5 choroid ECs were enriched in cell cycle- and chromosome-related transcripts, whereas P30 choroid ECs were enriched in adhesion- and ECM-related transcripts. (**g**) Pre-ranked GSEA using the GO term EXTRACELLULAR_MATRIX shows enrichment of ECM-related genes in P30 versus P5 choroid ECs. FDR, false discovery rate *q*-value; NES, normalized enrichment score; Nom. *p*, nominal *P* value. (**h**) Heatmap of the 79 genes included in the GSEA between P5 and P30 choroid ECs. Pearson correlation was used as a distance metric for hierarchical gene clustering. A blue-red colour scale depicts log2 transformed gene expression values (blue, low; red, high).

Because the oBRB does not become fully functional until choroid ECs complete their differentiation program^3^, we reasoned that comparing the transcriptomes of developing and adult choroid ECs would reveal gene sets specifically expressed in the latter that are necessary for the generation of mature RPE TJs. To this end, we isolated mouse choroid ECs to high purity at P5 (when rodent retina is undergoing terminal differentiation) and P30 (visual maturity) using a novel protocol that involves intravital staining of the specific EC marker VE-Cadherin followed by flow cytometry sorting^11^ (Fig. 1b, Supplementary Fig. 1). We extracted RNA immediately after sorting and carried out RNAseq analyses from three independent isolations (whole data set in Supplementary Data 1). Boxplots of log2 fragments per kilobase of transcript per million reads (FPKM) showed that the overall range and quartile distribution was consistent among samples (Fig. 1c), indicating that the results were reproducible and of high quality. Very low levels of contaminating non-ECs were present in the preparations (Fig. 1d). Hierarchical clustering analysis demonstrated that P5 and P30 choroid EC transcriptomes cluster separately (Fig. 1e), consistent with different overall functional phenotypes of immature and mature choroid ECs. Gene ontology analyses using DAVID software revealed that whereas the P5 choroid EC transcriptome was preferentially enriched in cell cycle- and chromosome-related transcripts, reflecting an immature phenotype, the transcriptome of adult (P30) choroid ECs was conspicuously enriched in genes encoding proteins involved in ‘biological adhesion', including a variety of extracellular matrix (ECM)-related genes (Fig. 1f and Supplementary Data 1). Indeed, cellular component ontology analysis demonstrated a highly significant increased expression of ECM-related genes in P30 compared to P5 choroid ECs (Fig. 1f and Supplementary Data 1). This result was confirmed by gene set enrichment analyses (GSEA; Fig. 1g,h). Collectively, these results strongly suggest that mature choroid ECs actively participate in ECM assembly and regulation.

### EC-secreted factors regulate RPE barrier function

**Figure 2:**
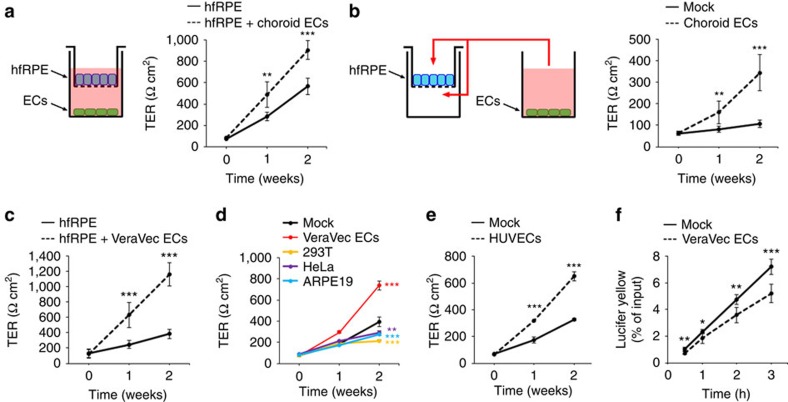
ECs increase TER and decrease paracellular permeability in hfRPE. (**a**) Co-culture of hfRPE with choroid ECs as depicted promotes an increase in hfRPE TER (*n*=6, *t-*test). *hfRPE versus hfRPE+choroid ECs at each time point. (**b**) Conditioned media from choroid ECs promotes an increase in hfRPE TER (*n*=6, *t*-test). *mock versus choroid ECs at each time point. (**c**) Co-culture of hfRPE with VeraVec ECs as depicted in **a** promotes an increase in hfRPE TER (*n*=7, *t*-test). *hfRPE versus hfRPE+VeraVec ECs at each time point. (**d**) Conditioned media from VeraVec ECs, but not from 293T, HeLa or ARPE19 cells, promotes an increase in hfRPE TER (*n*=3, analysis of variance (ANOVA)). *mock versus VeraVec ECs, 293T, HeLa or ARPE19 at 2 weeks. (**e**) Conditioned media from naïve HUVECs promotes an increase in hfRPE TER (*n*=3, *t*-test). *mock versus HUVECs at each time point. (**f**) Incubation of hfRPE with conditioned media from VeraVec ECs for 2 weeks decreases Lucifer Yellow paracellular permeability across hfRPE (*n*=6, *t*-test). *mock versus VeraVec ECs at each time point. Data are presented as mean±s.d.

RPE TJs are key components of the oBRB (ref. 3). Hence, to test our hypothesis that choroid ECs regulate oBRB maturation, we carried out *in vitro* co-culture experiments to investigate whether choroid ECs modulate the assembly of RPE TJs, using transepithelial electrical resistance (TER) as a readout of RPE barrier function. We cultured primary human fetal RPE (hfRPE) until full confluency on the top side of Transwell chambers, and then transferred them to new chambers with E4ORF1-expressing adult mouse choroid ECs present or not in the bottom compartment (Fig. 2a). The adenoviral gene E4ORF1 readily promotes the survival and the maintenance of the endothelial phenotype in the absence of serum or any added factors^12^ and allows the expansion of highly pure mouse choroid ECs (>95% CD31^+^/VE-Cadherin^+^; Supplementary Fig. 1). Strikingly, the presence of choroid ECs on the bottom side of the Transwell chamber elicited a marked increase in hfRPE TER (Fig. 2a). Choroid EC-conditioned medium caused a similar enhancement of RPE TER (Fig. 2b), suggesting that choroid ECs secrete factors that facilitate the maturation of RPE TJs. We obtained similar results when we used VeraVec ECs (primary human umbilical vein ECs (HUVECs) expressing E4ORF1), instead of mouse choroid ECs (Fig. 2c,d). Importantly, medium conditioned by non-EC types did not enhance hfRPE TER (Fig. 2d). Of note, medium conditioned by naive HUVECs also enhanced hfRPE TER (Fig. 2e), ruling out E4ORF1 expression as a cause of the observed phenomenon. Furthermore, exposure of hfRPE to EC-conditioned medium reduced their paracellular permeability (Fig. 2f). These data provide strong evidence that angiocrine factors secreted by both choroid and non-choroid ECs stimulate RPE barrier function.

### EC-secreted factors regulate RPE basement membrane assembly

**Figure 3:**
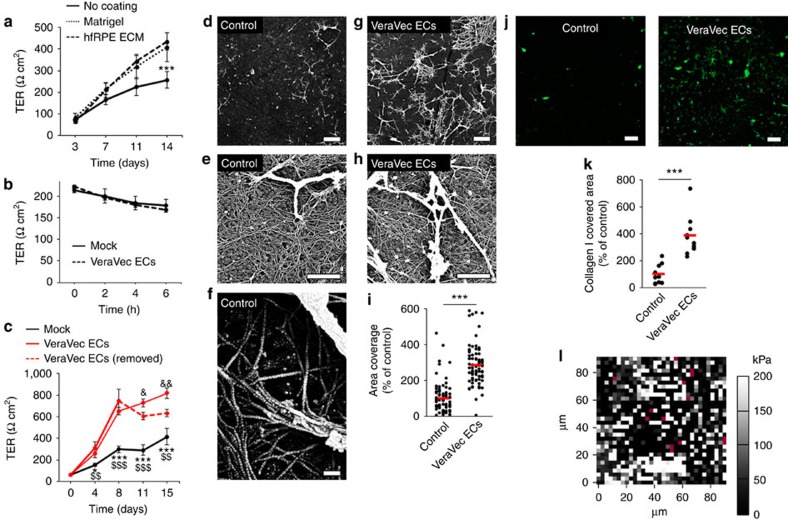
EC-secreted factors induce changes in hfRPE basement membrane. (**a**) hfRPE-derived ECM and Matrigel increase hfRPE TER. Transwell filters with hfRPE cultured for 5 months were decellularized, fresh hfRPE cells were seeded on top of hfRPE-derived ECM and TER was measured over time. As control, cells were plated on uncoated or Matrigel-coated Transwell filters (*n*=6, ANOVA). ***no coating versus Matrigel and hfRPE ECM at day 14. (**b**) Short time exposure of hfRPE (cultured for 12 days) to EC-conditioned media does not alter hfRPE TER (*n*=3, *t*-test). (**c**) Increased hfRPE TER values do not drop to control levels after removal of EC-conditioned media. EC-conditioned media was added to hfRPE cultures (continuous red lines). After 8 days, some cultures were kept in EC-conditioned media (continuous red line), whereas others were switched to mock conditioned media (dashed red line). *mock versus VeraVec ECs; ^$^mock versus VeraVec ECs (removed); ^&^VeraVec ECs versus VeraVec ECs (removed) (*n*=3, ANOVA). (**d**–**h**) EC-secreted factors enhance the formation of collagen-like bundles within hfRPE basement membrane. hfRPE were cultured in the absence (**d**–**f**) or presence (**g**,**h**) of VeraVec ECs as depicted in Fig. 2a. After 2 weeks in culture, hfRPE cells were removed by decellularization and filters were imaged by scanning electron microscopy. Bars: (**d**,**g**), 10 μm; (**e**,**h**), 1 μm; (**f**), 0.1 μm. (**i**) Bundles were quantified by analysing 90 images per condition from three biological replicates (*t*-test) with the magnification shown in **d** and **g**. Data are represented as the percentage of bundle-covered area relative to control conditions. (**j**) Immunofluorescence analyses of decellularized filters show the EC-mediated increased accumulation of collagen I-positive structures within hfRPE basement membrane. Bar, 10 μm. (**k**) Quantification of **j** (10 images from 2 biological replicates, *t*-test). (**l**) Atomic force microscopy showing the heterogeneous stiffness of hfRPE basement membrane. Shown is a representative image (*n*=3) of 90 μm × 90 μm with 1,024 individual points. A black-white colour scale represents increasing moduli of elasticity measured in kPa. Magenta pixels represent indeterminations. Median stiffness: 33 kPa. Data in **a**–**c** are presented as mean±s.d.

Next, we sought to gain insight into the underlying mechanisms mediating angiocrine stimulation of RPE barrier function. Since adult choroid ECs are enriched in ECM-related transcripts, we hypothesized that these mechanisms might involve regulation of the ECM. Indeed, fresh hfRPE plated on Transwell filters coated with either Matrigel or hfRPE-derived ECM displayed enhanced TER (Fig. 3a), consistent with a regulatory role of ECM on RPE barrier function. However, short exposure to EC-conditioned medium did not increase hfRPE TER (Fig. 3b), indicating that regulation by ECs is a slow and long-term process. Moreover, removal of EC-conditioned medium after stimulation of hfRPE TER did not return TER to basal levels (Fig. 3c), indicating that the regulatory effect, once installed, is stable. Scanning electron microscopy analysis of the ECM deposited on filters by hfRPE revealed a uniform mesh of thin collagen fibres, sprinkled with occasional thick collagen bundles (Fig. 3d–f). Similar collagen bundles have been described in the inner collagen layer of the RPE basement membrane *in vivo*^13^,^14^. Strikingly, such bundles were significantly more abundant when hfRPE had been co-cultured with ECs (Fig. 3g–i, Supplementary Fig. 2a). Immunofluorescence analyses of decellularized filters demonstrated collagen I-positive structures corresponding to the collagen bundles observed by electron microscopy, which were clearly more abundant in the presence of EC-conditioned medium (Fig. 3j,k) without an associated increase in hfRPE-expressed collagen I mRNA (Supplementary Fig. 2b). Of note, both electrical resistance and Lucifer Yellow flux across decellularized filters were the same regardless of whether hfRPE had been cultured in the absence or presence of ECs (Supplementary Fig. 2c,d), indicating that TER and permeability changes mediated by EC-conditioned medium were dependent on the presence of hfRPE and not on the abundance of collagen bundles *per se*. To characterize the biomechanical aspects of these collagen structures, we carried out atomic force microscopy to measure the stiffness of hfRPE-deposited ECM. The deposited ECM had a median modulus of elasticity (*E*) of ∼30 kPa but was highly heterogeneous at the micrometre range, with spots over 5-fold stiffer appearing with roughly the same spatial frequency as the collagen bundles we observed by electron microscopy and immunofluorescence assays (Fig. 3l). Taken together, our experiments suggest a model in which ECs regulate RPE barrier function by secreting factors that promote the assembly of stiffer collagen bundles in RPE's basement membrane.

### Role of lysyl oxidases in BM assembly and visual function

**Figure 4:**
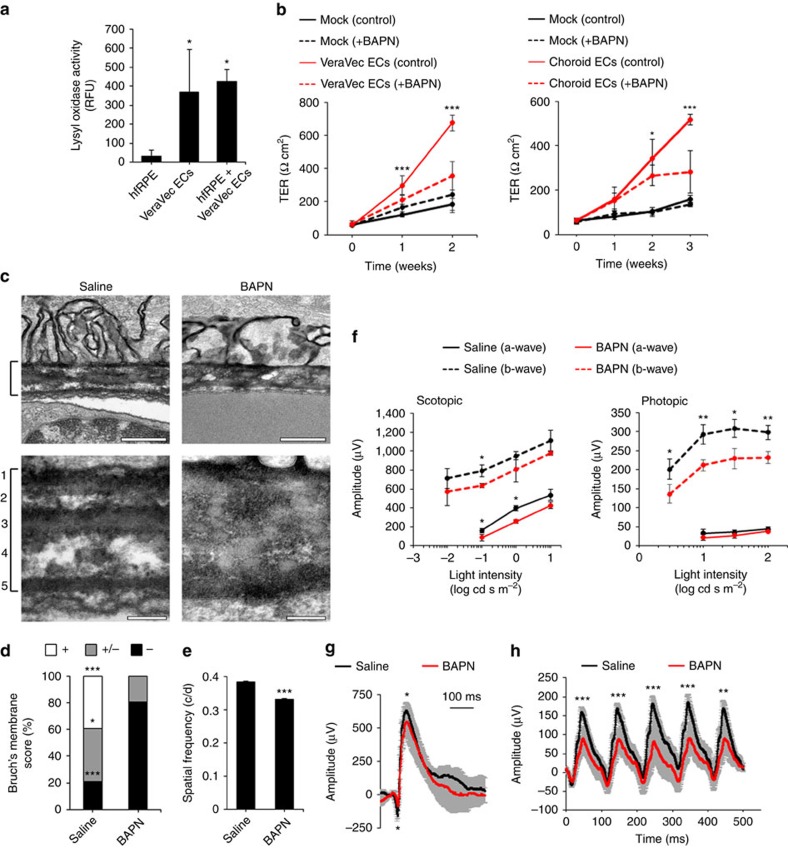
Inhibition of lysyl oxidase activity impairs EC-mediated increase in hfRPE TER and alters BM assembly and visual function. (**a**) Lysyl oxidase activity in the basolateral medium of hfRPE cultures is increased in the presence of ECs. hfRPE, VeraVec ECs or both were (co-) cultured for 1 week in Transwell inserts as depicted in Fig. 2a. Media was collected from the basolateral compartment and lysyl oxidase activity was determined (*n*=3, ANOVA). Data are presented as relative fluorescence units (RFU). *VeraVec ECs and hfRPE+Veravec ECs versus hfRPE. (**b**) Lysyl oxidase inhibition impairs EC-mediated increase in hfRPE TER. hfRPE cells were cultured in the absence or presence of media conditioned by VeraVec ECs (left, *n*=6) or choroid ECs (right; 0, 1 and 2 weeks, *n*=6; 3 weeks, *n*=3, ANOVA), supplemented or not with the lysyl oxidase activity inhibitor BAPN (300 μM), and TER was measured over time. For clarity, only the statistical analyses of VeraVec/choroid ECs control versus BAPN are shown. (**c**) BM 5-layered ultrastructure (bracket, numbered 1–5) of P30 mice is altered after inhibition of lysyl oxidase activity *in vivo* during terminal retinal differentiation as analysed by transmission electron microscopy. Bars: top panels, 500 nm; bottom panels, 100 nm. (**d**) Quantification of (**c**) (saline, 38 images from *n*=4; BAPN, 47 images from *n*=4, N-1 *χ*^2^ test). *saline versus BAPN. (**e**) Optomotor response analyses of P30 mice treated as in **c** showed that BAPN induced a significant reduction in visual function (*n*=4, *t*-test). c/d, cycles per degree. (**f**) ERG assays of P30 mice treated as in **c** show BAPN-induced reduction in a-wave (scotopic ERG) and b-wave (scotopic and photopic ERG) amplitude (*n*=3, *t*-test). *saline versus BAPN (either a- or b-wave) for each light intensity. (**g**) Example of a scotopic ERG corresponding to 0.1 cd s m^−2^ light intensity shown in **f** (*n*=3, *t*-test). *saline versus BAPN (a- and b-wave). (**h**) Flicker response of P30 light-adapted mice treated as in (**c**) (*n*=3, *t*-test). *saline versus BAPN at each peak. Data in **a**,**b**,**e**–**h** are presented as mean±s.d.

A search for factors that might be mediating such a phenomenon led us to lysyl oxidases, a family of enzymes that catalyse elastin and collagen crosslinking and are essential for ECM assembly^15^. We found that supernatants from ECs and RPE-EC co-cultures contained much higher lysyl oxidase activity than hfRPE cultured alone (Fig. 4a). We observed that expression of lysyl oxidases in hfRPE was not significantly altered by EC-secreted factors (Supplementary Fig. 3a), suggesting that the increased lysyl oxidase activity detected in the basolateral medium of hfRPE-EC co-cultures derived mainly from ECs. Remarkably, inhibition of lysyl oxidase activity by addition of the specific inhibitor β-aminopropionitrile (BAPN) to EC-conditioned media impaired the EC-mediated increase in hfRPE TER (Fig. 4b) without compromising hfRPE viability (Supplementary Fig. 3b). Noteworthy, BAPN treatment of fully polarized hfRPE did not reduce TER (Supplementary Fig. 3c), showing that inhibition of lysyl oxidase activity effectively reduces the EC-elicited TER increase during the assembly but not after full maturation of RPE TJs.

BM is a highly organized, five-layered basement membrane that separates RPE from the choroid vasculature^2^. To test for the possible role of lysyl oxidase activity in BM assembly, we performed daily BAPN intraperitoneal injections into mice during the first month after birth. Transmission electron microscopy images clearly showed that lysyl oxidase inhibition resulted in a disorganized BM, lacking its characteristic five-layer structure (Fig. 4c,d and Supplementary Fig. 4). Importantly, these structural changes were paralleled by functional vision defects: BAPN treatment induced a reduction of spatial visual function measured by optomotor response assays^16^ (Fig. 4e) and a decrease in the amplitude of the a-wave, b-wave (Fig. 4f,g) and flicker response (Fig. 4h) in electroretinogram (ERG) assays. With the exception of the aforementioned BM alterations, we did not detect any BAPN-induced changes in overall retinal structure (Supplementary Fig. 3d,e), indicating that impaired visual function was likely caused by subtle alterations of PR function rather than by PR loss and major disruption of retinal architecture. These results strongly suggest that lysyl oxidases play a key role in the maturation of BM, which in turn is required for the development of normal visual function.

### Mechanisms involved in EC-mediated RPE TJ regulation

**Figure 5:**
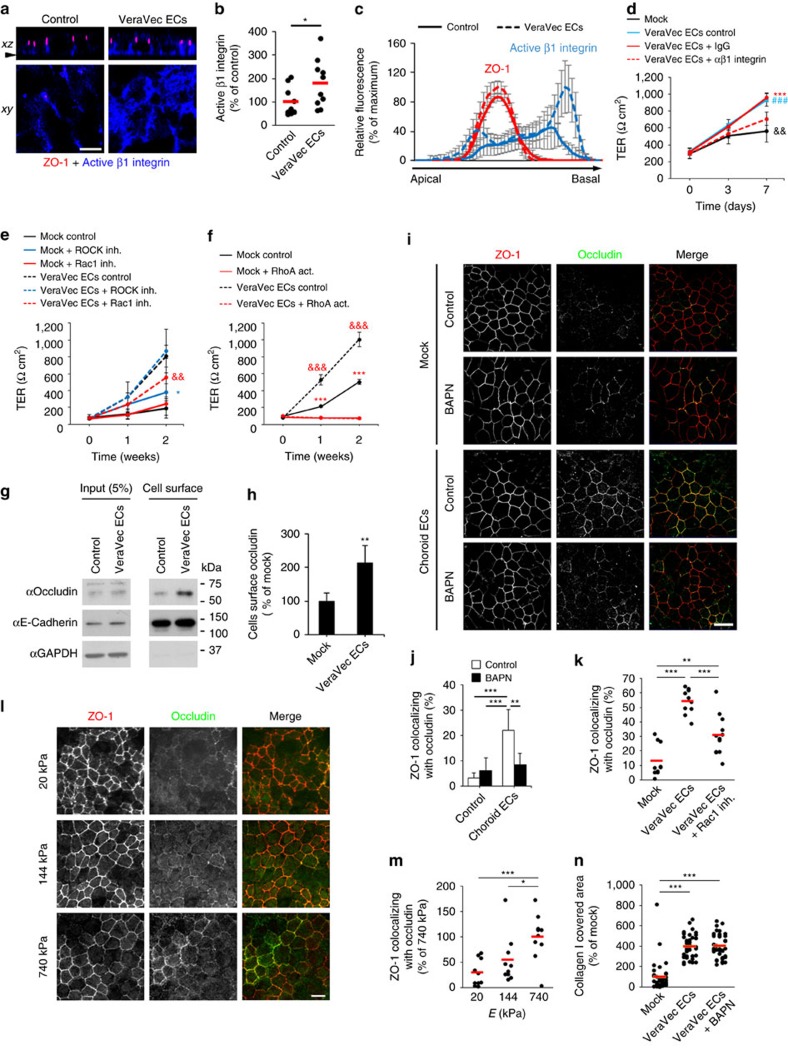
Molecular mechanisms involved in EC-mediated regulation of hfRPE TJs. (**a**–**c**) ECs increase the levels of active β1 integrin in hfRPE basal plasma membrane. (**a**,**b**) Active β1 integrin (blue) and the TJ protein ZO-1 (red) in hfRPE were assessed by immunofluorescence assays (10 z-stacks from 2 biological replicates, *t*-test). Top panel, orthogonal view (xz) of a confocal z-stack. Bottom panel, confocal plane (xy) indicated by the arrowhead in the top panel. Bar, 10 μm. (**c**) Example of the relative fluorescence of individual confocal planes from 5 z-stacks is shown from the most apical (left) to the most basal (right) planes. (**d**) Inhibition of hfRPE β1 integrin with a blocking antibody impairs EC-mediated increase in TER. For clarity, only the statistical analyses at day 7 are shown (*n*=6, ANOVA), all groups versus VeraVec ECs+anti-β1 integrin). (**e**,**f**) RhoA/ROCK and Rac1 pathways are involved in the EC-mediated increase in hfRPE TER. For clarity in **e**, only the statistical analyses at week 2 are shown. In **e**: *Mock control versus mock+ROCK inhibitor; ^&&^VeraVec ECs control versus VeraVec ECs+Rac1 inhibitor (*n*=6, ANOVA). In **f**: ***Mock control versus mock+RhoA activator; ^&&&^VeraVec ECs control versus VeraVec ECs+RhoA activator (*n*=3, ANOVA). (**g**,**h**) Cell surface biotinylation assays showing EC-mediated increase in occludin cell surface localization in hfRPE (*n*=4, *t*-test). (**i**,**j**) Immunofluorescence assays show a lysyl oxidase activity-dependent enhancement of occludin accumulation (green) along ZO-1-positive TJs (red) in hfRPE when exposed to choroid EC-conditioned media (*n*=5, ANOVA). Bar, 20 μm. (**k**) Rac1 inhibition impairs EC-mediated occludin accumulation at hfRPE TJs [10 (mock and VeraVec ECs) or 12 (VeraVec ECs+Rac1 inhibitor) z-stacks from 2 biological replicates, ANOVA). (**l**,**m**) Increasing substrate stiffness enhances occludin localization at hfRPE TJs (10 z-stacks from 2 biological replicates, ANOVA). Bar, 10 μm. (**n**) Inhibiton of lysyl oxidase acitivity does not impair EC-mediated accumulation of collagen I in hfRPE basement membrane (40 images per condition from 2 biological replicates, ANOVA). Data in **c**–**f**,**h** and **j** are presented as mean±s.d.

We also found that exposure to EC-conditioned medium increased the levels of active β1 integrin in hfRPE (Fig. 5a,b), specifically at the basal plasma membrane (Fig. 5c and Supplementary Fig. 5a). Importantly, inhibition of hfRPE β1 integrin with a blocking antibody impaired EC-mediated increase in hfRPE TER (Fig. 5d). These findings support the participation of β1 integrin receptors in cellular signalling processes activated by changes in physical properties of the ECM^17^,^18^. Reportedly, β1 integrin signalling modulates pathways regulated by the small GTPases Rac1 and RhoA (refs 19, 20, 21). In the absence of EC-conditioned media, addition of the Rac1 inhibitor NSC 23766 did not alter the progressive hfRPE TER increase; however, the ROCK inhibitor Y-27632 increased TER values (Fig. 5e). On the other hand, whereas ROCK inhibition did not impair or improve the EC-mediated hfRPE TER increase, Rac1 inhibition significantly abrogated such effect (Fig. 5e). These results suggest that EC conditioned medium induces an increase in hfRPE TER at least in part by activating Rac1 signalling and inhibiting the ROCK pathway. Indeed, RhoA activation by treatment with CN03 totally blocked hfRPE TER development in the absence or presence of EC-conditioned medium (Fig. 5f). Moreover, whereas EC-conditioned medium did not alter the localization of the TJ protein claudin-19, the most abundant claudin in hfRPE^22^, it promoted the accumulation of occludin at the surface of hfRPE cells (Fig. 5g,h and Supplementary Fig. 5b) specifically along narrow areas where it co-localized with the TJ marker ZO-1 (Fig. 5i,j and Supplementary Fig. 5c,d). This accumulation occurred in a lysyl oxidase activity- and Rac1-dependent manner (Fig. 5i–k and Supplementary Fig. 5e). Remarkably, accumulation of occludin at ZO-1-positive areas increased when hfRPE cells were cultured on top of polyacrylamide gels of increasing stiffness (Fig. 5l,m). Interestingly, inhibition of lysyl oxidase activity did not affect EC-mediated accumulation of collagen I-positive structures within hfRPE basement membrane (Fig. 5n and Supplementary Fig. 5f). This result suggests a role of additional EC-secreted factors other than lysyl oxidases in RPE basement membrane assembly, and underscores the importance of ECM crosslinking (and not only ECM deposition) in EC-mediated regulation of hfRPE barrier function. Surprisingly, BAPN treatment resulted in a further increase in the levels of active β1 integrin at hfRPE basal plasma membrane induced by EC-conditioned medium (Supplementary Fig. 5g). These data highlight the complexity and fine tuning of the mechanisms involved in ECM-mediated regulation of RPE barrier function, and suggest that accumulation of defectively crosslinked ECM may over-activate β1 integrin signalling and impair proper RPE TJ assembly. Taken together, our results suggest that EC-secreted factors increase the frequency of stiffer anchor points in RPE basement membrane that, on sensing by RPE β1 integrin, trigger Rac1 and RhoA/ROCK pathways that modulate the function and molecular composition of RPE TJs.

## Discussion

We report the transcriptome analysis of native choroid ECs obtained from both developing and adult mouse eyes. To our knowledge, this is the first study reporting the gene expression profile of tissue-specific ECs isolated at different developmental stages. A key result of the study was that during terminal retinal differentiation, choroid ECs significantly increase the expression of structural and regulatory ECM genes. Whether this is a choroid EC-specific phenomenon or a general feature of differentiating ECs elsewhere is not known. Our *in vitro* and *in vivo* studies support a model in which choroid ECs regulate the assembly of the RPE basement membrane, which in turn enhances the oBRB by regulating RPE TJ maturation and barrier function. The regulation of hfRPE TER by EC-conditioned medium was selectively observed for both human umbilical vein and mouse choroid ECs but not for non-EC cells, suggesting that it may occur not exclusively in the eye but, rather, throughout the organism. Indeed, there is some evidence of EC-mediated TJ regulation in the airway epithelium^23^,^24^ and kidney proximal tubule^25^. Furthermore, previous publications support a central role of the ECM in regulating epithelial cell–cell junctions, for example, in the blood-testis barrier^26^, glomerular filtration barrier^27^ and choroid plexus^28^,^29^. Our study goes beyond these findings in that it implicates ECs in such process, establishing a connection between EC-secreted molecules, basement membrane stiffness and epithelial TJs. This may be an important finding for the retinal disease field, because the choroidal vasculature is known to undergo progressive atrophy in patients affected by retinal pathologies such as age-related macular degeneration (AMD), a devastating incurable human disease^30^. Of note, changes in BM stiffness and choroidal ECM turnover have been also associated with aging and/or AMD^31^, and GWAS studies have identified several ECM-related genes as susceptibility loci for AMD^32^,^33^. In this context, it is plausible that defects in choroid ECs could alter their ability to regulate the assembly of the RPE basement membrane, contributing to the structural and homeostatic alterations observed in aged and diseased retinas. Indeed, it is likely that EC-mediated ECM modulation may affect oBRB barrier function by regulating not only RPE TJs, but also the polarized distribution of membrane transporters^34^,^35^ and the activity of mechanosensitive ion channels^36^,^37^. Both features are key for membrane ion flux and the formation of osmotic gradients, essential components of transepithelial transport^4^. Hence, defective choroid ECs could induce the alteration of RPE-mediated bidirectional transport between the subretinal space and choroidal circulation, leading to some of the features observed in the outer retina of aged subjects and AMD patients.

Our data are consistent with previous studies in other biological systems reporting a connection between ECM stiffness and β1 integrin activation^17^, the role of β1 integrin in the acquisition of epithelial polarity^19^,^20^ and the antagonistic effects of Rho/ROCK and Rac1 pathways on cell–cell adhesion^38^,^39^. The specific role of occludin at TJs is not fully understood, since occludin knockout animals develop apparently normal TJs but exhibit phenotypes compatible with indirect effects on permeability regulation^40^. Indeed, both *in vitro*^41^,^42^,^43^,^44^ and *in vivo*^45^,^46^ studies have demonstrated a key role of occludin in epithelial barrier function. Thus, occludin levels in RPE TJs may be an important determinant of normal or pathological oBRB regulation.

Stem cell-derived RPE transplantation has emerged as a potential therapeutic intervention to replace lost RPE cells in AMD patients^47^. A limitation of this approach is that survival and function of transplanted RPE can be compromised by a damaged BM in the diseased eye. *In vitro* experiments have shown that RPE survival on human BM explants from aged or AMD patients is markedly increased after adding EC-conditioned medium to the cultures^48^. Furthermore, the ability of RPE to phagocytose rod outer segments *in vitro* (a normal circadian function of RPE cells that promotes PR renewal) is impaired when cells are grown on aged BM explants, and can be improved by recoating the explants with ECM proteins^49^,^50^. Our results, together with these studies, strongly suggest that cell-ECM interactions are key not only to promote RPE survival, but also to enable RPE differentiated functions. Hence, our observations may have translational applications as they suggest that EC-secreted factors or chemical analogues might be used to enable or accelerate the generation of stem cell-derived RPE with a mature structural and functional RPE phenotype.

In conclusion, our studies support the existence of a novel signalling pathway for regulation of the oBRB. Maturing choroid ECs increase the expression of ECM genes and secrete angiocrine factors, resulting in a remodelled RPE basement membrane; RPE integrin receptors sense these changes, triggering Rho GTPase signals that enhance RPE barrier function. Albeit our experiments focused on RPE TJs, future work will likely show a more comprehensive modulatory role of choroid ECs on the oBRB by regulating RPE polarity and directional ion and water transport. Our results predict similar regulatory roles of ECs in parenchymal cell barrier function in other body organs. Current research in our laboratory focuses on various aspects of EC-RPE crosstalk and their impact on the onset and progression of retinal diseases such as AMD.

## Methods

### Mouse choroid EC isolation

Mouse choroid ECs were isolated following a previously described protocol to isolate mouse ECs (ref. 11) with minor modifications. Briefly, 25 μg (100 μl) Alexa Fluor 647-conjugated anti-mouse VE-Cadherin (clone BV13, cat. 138006, BioLegend Inc.) was injected retro-orbitally using a 30G needle into male and female adult (P30) 129/B6 mice under anaesthesia. P5 mice were anaesthetized by hypothermia and received 12.5 μg (50 μl) antibody retro-orbitally using a 31G needle attached to a 0.3 ml insulin syringe (BD Biosciences). After 10 min, mice were euthanized and the eyeballs were enucleated followed by careful excision of any extraocular tissue that remained attached to the sclera. The anterior segment was discarded, and after removal of the neural retina, RPE/choroid was mechanically dissected from the sclera using a scalpel. Isolated RPE/choroid tissues from 14 eyes were pooled for each preparation and incubated at 37 °C in serum-free DMEM supplemented with 4 × digestion buffer: 25 mg ml^−1^ collagenase A, 25 mg ml^−1^ dispase II, 250 μg ml^−1^ DNase (Roche), 140 mM NaCl, 5 mM KCl, 2.5 mM phosphate buffer, 10 mM HEPES, 2 mM CaCl_2_ and 1.3 mM MgCl_2_. After 10 min, tissue was pipetted several times to facilitate digestion and incubated at 37 °C for 5 more minutes. Cells were filtered through a 40 μm strainer to remove undigested tissue, pelleted and resuspended in ice-cold serum-free DMEM. After a second filtration through a 35 μm strainer, VE-Cadherin+ cells were sorted by flow cytometry using a FACSJazz (BD Biosciences). This protocol was reviewed and approved by the Institutional Animal Care and Use Committee.

### Cell culture

To establish mouse choroid EC cultures, sorted cells (∼50,000 per sorting) from P30 mice were seeded in fibronectin-coated (EMD Millipore) 24-well plates and cultured at 37 °C, 5% CO_2_ and 5% O_2_ in complete mouse EC media: Advanced DMEM/F12 media (Life Technologies), 50 μg ml^−1^ endothelial cell supplement (Biomedical Technologies), 20% fetal bovine serum (FBS) (Omega Scientific), antibiotic-antimycotic 100 × -solution (Invitrogen), 10 mM HEPES (Invitrogen), 5 μM SB431542 (R&D), 50 μg ml^−1^ heparin (Sigma), Glutamax 100 × -solution, MEM non-essential amino acids 100 × -solution (Life Technologies), 20 ng ml^−1^ FGF-2 and 10 ng ml^−1^ VEGF (Peprotech). Two days after isolation, cells were infected with E4ORF1-coding lentivirus (Angiocrine Bioscience; multiplicity of infection ∼5) and expanded. At passage 2, VE-Cadherin^+^/CD31^+^ ECs were re-purified by flow cytometry (2.5 μg per sample, anti-VE-cadherin; 2.5 μg per sample, anti-CD31, clone 390, cat. 11-0311-82, eBioscience) and further expanded to obtain necessary cell numbers for experiments.

hfRPE cells from donors at 16 to 18 weeks of gestation were kindly provided by S. Miller and cultured at 37 °C, 5% CO_2_ in RPE medium^51^ (see section below for details on cell culture during TER assays): MEM, α modification containing N1 supplement 100X-Solution, hydrocortisone (20μgl^−1^), taurine (250μgl^−1^), triiodo-thyronin (0.013μgl^−1^) (Sigma), Penicillin-Streptomycin 50X-solution (Corning), 5% fetal bovine serum, Glutamax 100X-solution and MEM non-essential amino acids 100X-solution (Life Technologies). Cells were used in passage 0 or 1. The RPE cell line ARPE-19 (ATCC) was cultured in the same medium as hfRPE. 293T and HeLa cells (ATCC) were cultured in DMEM (Corning), 10% fetal bovine serum (Life Technologies) and Penicillin-Streptomycin 50X Solution (Corning). None of these cell lines are listed in the database of commonly misidentified cell lines maintained by ICLAC. Cell lines were not authenticated. Human umbilical vein ECs (HUVECs) and VeraVec ECs (E4ORF1-expressing HUVECs) 12 (provided by Angiocrine Bioscience) were cultured at 37°C, 5% CO_2_ in EC growth medium: M199 (Hyclone), 50μgl^−1^ endothelial cell supplement (Biomedical Technologies), 20% fetal bovine serum (Life Technologies), Penicillin-Streptomycin 50X-solution (Corning), 10 mM HEPES (Invitrogen), 50μgl^−1^ heparin (Sigma) and Glutamax 100X-solution (Life Technologies). Cell cultures were not routinely tested for mycoplasma infection.

### RNAseq analyses

For RNAseq of native choroid ECs from P5 and P30 mice, RNA was extracted immediately after cell sorting by lysing cells directly in 750 μl TRI Reagent (Molecular Research Center, Inc.) and following the manufacturer's instructions. After phase separation, the aqueous phase was diluted 1:1 with 70% ethanol and loaded into a column of the RNeasy Mini Kit (Qiagen). RNA was purified following the manufacturer's instructions, including an in-column DNAse digestion step. RNAseq was carried out from three independent isolations, with 14 eyes per isolation. cDNA libraries were prepared with the TruSeq RNA Sample Preparation Kit (Illumina). Four samples were run per lane and sequenced on an Illumina HiSeq2000 platform. On quality control using FastQC, raw reads were aligned to the mouse genome (mm9) using TopHat with default parameters. CuffLinks with GC and upper quartile normalization was then used to calculate normalized expression levels (FPKM; Fragments Per Kilobase of transcripts per Million reads)^52^. Log transformed FPKM profiles were clustered using hierarchical clustering (hclust function in the R language) with average linkage and one minus Pearson correlation as distance. To determine the genes differentially expressed between P5 and P30 choroid ECs, statistical significance was calculated using DEseq2 bioconductor package in R (ref. 53), which uses read counts (htseq-count). Only genes with Benjamini–Hochberg corrected *P*<0.01 and with a minimum relative difference of 2-fold were considered. All FPKM values were added 1 before filtering to avoid losing genes whose expression was undetectable in P5 or P30 samples. Next, the lists of upregulated genes in either P5 or P30 were re-filtered to exclude genes with FPKM<5 (close to the detection limit). These lists of differentially expressed genes were subsequently used to carry out gene ontology analyses with the DAVID software^54^, using the biological process (GOTERM_BP_FAT), cellular component (GOTERM_CC_FAT) and molecular function (GOTERM_MF_FAT) categories. GSEA^55^ using the term EXTRACELLULAR_MATRIX (GO term GO:0031012) were performed to compare the transcriptomes of P5 and P30 choroid ECs.

### Co-culture assays and TER measurements

2 × 10^5^ hfRPE cells were seeded on polyester membrane Transwell inserts (12 mm diameter, 0.4 μm pore; Corning) in RPE medium supplemented with 5% FBS and 10 μM Y-27632 (Tocris) to minimize dedifferentiation^56^. Unless otherwise indicated, cells were seeded on uncoated Transwell inserts (that is, without ECM coating). Where indicated, Transwell inserts were coated with growth factor-reduced Matrigel (BD Biosciences; 1:50 dilution). After culturing cells for 3 days to ensure total confluency, media was switched to RPE medium supplemented with 1% FBS (RPE 1% FBS) and this was considered day 0. For co-culture experiments, choroid ECs or VeraVec ECs were cultured in 12-well plates until confluency (see cell culture section above). At day 0, media was changed to RPE 1% FBS and cells were co-cultured with hfRPE grown in the top chamber of Transwell inserts. Media was changed every 3–4 days. Conditioned media was generated by culturing ECs, 293T, HeLa and ARPE19 cells in RPE 1% FBS medium for 48 h. Media were cleared by centrifugation (400 g, 3 min) and kept at −80 °C in working aliquots until further use. Conditioned media were added to hfRPE cultures at day 0 as described above. Where indicated, 300 μM BAPN (Sigma), 10 μM Y-27632 (ref. 57), 100 μM NSC 23766 (ref. 58; Tocris) or 1 μg ml^−1^ CN03 (Cytoskeleton) was added to mock or EC-conditioned media, which were changed every 3–4 days. Where indicated, medium in the bottom chamber of Transwell inserts was supplemented with 8 μg ml^−1^ control rat IgG1 (clone HRPN, cat. MABF1786) or blocking anti-β1 integrin antibody (clone AIIB2 (ref. 20), cat. MABT409) (EMD Millipore). TER was measured in triplicate with an EVOM Voltohmeter (World Precision Instruments) before changing media, and TER values were expressed in ohms (Ω) cm^2^ after background subtraction.

### Cell viability assays

Cells cultured in Transwell inserts were incubated with 10% Alamar Blue (Invitrogen) in Optimem (Thermo Fisher Scientific), 0.25 ml and 0.75 ml in the upper and lower chamber, respectively. After 2 h at 37 °C, media from both chambers were collected and mixed. Absorbance was measured at 570 and 600 nm and calculations were carried out according to the manufacturer's instructions.

### Paracellular permeability assays

hfRPE were cultured in Transwell inserts for 2 weeks in the presence of mock or VeraVec EC-conditioned media. Permeability assays were carried out with intact cultures or decellularized inserts. After washing with phenol red-free, Ca- and Mg-supplemented HBSS buffer (Ca/Mg HBSS; Corning), 100 μM Lucifer Yellow lithium salt (457.2 Da; Molecular Probes) in Ca/Mg HBSS was added to the top chamber of the insert (0.5 ml) and 1 ml Ca/Mg HBSS was added to the bottom chamber. 50 μl aliquots were removed from the bottom chamber after 0.5, 1, 2 and 3 h of incubation at 37 °C and fluorescence was measured in a SpectraMax M2 microplate reader (Molecular Devices; excitation, 485 nm; emission, 530 nm) along with a standard curve. Data are presented as percentage of the input.

### Culture decellularization

hfRPE cells were cultured for the indicated times on Transwell inserts or coverslips. After washing with PBS, cells were incubated in PBS containing 0.5% Triton X-100 and 20 mM NH_4_OH for 5 min at 37 °C (ref. 59). Decellularized cultures were washed 5 times in PBS and used for further experiments.

### Electron microscopy

For scanning electron microscopy, hfRPE cultures in Transwell inserts (2 weeks) were decellularized, fixed for 1 h at room temperature in 0.1 M sodium cacodylate buffer containing 2.5% glutaraldehyde, 4% paraformaldehyde and 0.02% picric acid (fixation buffer) and post-fixed for 1 h at room temperature in an aqueous solution of 1% OsO_4_ and 1.5% K-ferricyanide (post-fixation buffer). Samples were dehydrated through graded ethanol series up to absolute ethanol, critical point dried through liquid CO_2_, mounted on aluminium stubs and sputter coated with gold-palladium. Imaging was carried out on a Zeiss Leo 1550 scanning electron microscope using Zeiss InLens and SE2 secondary electron detectors. Images were automatically thresholded and quantified with ImageJ software.

For transmission electron microscopy, mouse eyes were enucleated, washed in Ca/Mg HBSS and incubated at room temperature in fixation buffer. After 10 min, a hole was made in the cornea with a 22G needle and incubated overnight at 4 °C in the same buffer. After washing in 0.1 M sodium cacodylate buffer, the cornea and lens were removed from each eye. After incubation in post-fixation buffer for 2 h at room temperature, eyes were *en bloc* stained in 1.5% uranyl acetate in water for 2 h at room temperature in the dark and dehydrated through graded ethanol series up to absolute ethanol. After transitioning through ethanol-acetonitrile and acetonitrile-resin mixtures, the eyes were infiltrated under light vacuum and embedded in LX-112 resin (Ladd Research Industries). Blocks were polymerized at 50 °C for 36 h and cut with a jeweler's saw to expose the midpoint of the eye and to re-orient the block for sectioning. 70 nm-thick sections were mounted on 200 mesh thin-bar copper grids and contrasted with lead citrate. Samples were imaged in a JEOL JSM-1400 transmission electron microscope operated at 100 kV and images were captured on a Veleta 2 k × 2 k CCD camera (Soft Imaging Solutions). To quantify BAPN-induced BM alterations, we imaged multiple BM regions (1 μm wide, 6–12 images per animal) and scored them as follows: +, evident 5-layered structure; ±, partial 5-layered structure (discontinuous or faint layers); −, no detectable 5-layered structure.

### Atomic force microscopy

After 7 weeks in culture on coverslips, hfRPE cultures were decellularized. An MFP-3D-Bio atomic force microscope (Asylum Research) was utilized to take force maps of 90 × 90 μm. Each of the force maps was made up of a 32 × 32 grid of individual force curves which were acquired using a trigger point of 20 nN and a force distance of 4 μm with a velocity of 4 μm s^−1^. Force curves were acquired with a polystyrene, 6.1 μm diameter CP-Cont-PS colloidal probe (sQube) and fitted with the Hertz model. Asylum Research's software (Igor Pro 6.34A) was used for the thermal tuning calibrations of the cantilever spring constant.

### Lysyl oxidase activity assays

hfRPE cells were cultured in RPE 1% FBS medium for 1 week in the top chamber of Transwell inserts in the absence or presence of VeraVec ECs on the bottom chamber. VeraVec ECs cultured alone were also included in the assays. Conditioned media from the last 4 days were collected from the bottom chambers and lysyl oxidase activity was assessed with the lysyl oxidase activity assay kit (Abcam) following the manufacturer's recommendations. Parallel samples were assayed in the presence of 300 μM BAPN. Relative fluorescence units specifically derived from lysyl oxidase activity were calculated by subtracting the values obtained in the presence of BAPN to the ones obtained in its absence.

### Immunofluorescence assays

hfRPE cells were cultured on Transwell inserts for 2 weeks in the absence or presence of ECs or EC-conditioned media as shown in Fig. 2. hfRPE cells were rinsed with PBS, fixed in 4% paraformaldehyde for 10 min at room temperature, washed with 50 mM NH_4_Cl in PBS and permeabilized with 0.1% IGEPAL CA-630 (Sigma) in PBS for 10 min at room temperature. Insert membranes were excised from the inserts and blocked with 3% BSA in PBS at room temperature for 15 min. Cells were incubated for 1 h at 37 °C with mouse antibodies against active β1 integrin (1:200, clone HUTS-21 (ref. 60), cat. 556048, BD Pharmingen), occludin (1:100, clone OC-3F10, cat. 33–1500, Invitrogen) or claudin-19 (1:100, clone 2F2, cat. H00149461-M02; Novus Biologicals) in combination with rabbit anti-ZO-1 (1:200, cat. 40–2200, Invitrogen) diluted in 1% BSA in PBS. After washing in PBS, cells were incubated for 30 min at room temperature with secondary goat antibodies labelled with Alexa Fluor 488 or 568 (1:500, Life Technologies). Cells were washed three times in PBS and mounted in Vectashield (Vector Laboratories). Decellularized Transwell filters were processed the same way using a polyclonal collagen I antibody (1:200, cat. ab34710, Abcam). For cells grown on polyacrylamide gels inside Transwell inserts, insert membranes were removed before the blocking step and the protocol was continued just with the cells on the gels, mounting them in FluorSave reagent (Calbiochem). Mouse choroid ECs were cultured in gelatin-coated μ-Slide 8 Well ibiTreat chambers (Ibidi), fixed and stained with anti-CD31 antibody (1:100). Images were collected with a Zeiss Axio Observer spinning disk confocal microscope equipped with a Yokogawa scanner unit, Hamamatsu Evolve electron-multiplying charge-coupled device cameras (Photometrics) and Zeiss Plan Apochromat 63 × /1.46–0.60 oil immersion objective. For co-localization assays, image quantification was performed with Zen (Zeiss) software, and co-localization was quantified by calculating the Manders' coefficient for all the sections of a confocal stack. All images show a single confocal plane at the level of maximum ZO-1 intensity, except collagen I and hydrogel immunofluorescence assays (maximum intensity projection). The relative area covered by collagen I on decellularized Transwell inserts was quantified with ImageJ software after automatic threshold.

### Cell surface biotinylation and western blot

hfRPE were cultured for 2 weeks on Transwell inserts in the absence or presence of VeraVec ECs as depicted in Fig. 2a. hfRPE-containing inserts were transferred to a clean multiwell plate, rinsed three times in cold Ca/Mg HBSS and incubated for 20 min at 4 °C with 0.5 mg ml^−1^ Sulfo-NHS-SS-Biotin (Thermo Fisher Scientific) in Ca/Mg HBSS added to both chambers of the insert. After repeating the biotinylation step, cells were washed three times in 50 mM NH_4_Cl in Ca/Mg HBSS and lysed in 0.5 ml lysis buffer containing 1% Triton X-100, 0.1% SDS, 40 mM Tris-HCl (pH 7.6), 150 mM NaCl and protease inhibitor cocktail III (EMD Millipore) for 30 min at 4 °C. Lysates were cleared by centrifugation (15 min, 13,000 r.p.m., 4 °C) and 50 μl were combined with 50 μl 2 × Laemmli buffer for input determination. The rest of the lysate was incubated overnight with 50 μl NeutrAvidin agarose (Thermo Fisher Scientific) at 4 °C with rotation. Samples were washed three times with ice-cold lysis buffer, resuspended in Laemmli buffer and boiled for 10 min. Bound proteins were separated on SDS-polyacrylamide gels and analysed by western blot using rabbit anti-occludin (1:1,000, cat. 71–1,500, Thermo Fisher Scientific), mouse anti-E-cadherin (1:1,000, clone HECD-1, cat. 13–1,700, Thermo Fisher Scientific) and chicken anti-GAPDH (1:10,000, cat. GW22763, Sigma). Band detection was carried out with ECL (GE Healthcare) or the Odyssey system (LI-COR Bioscience). Quantification of cell surface occludin was carried out with ImageJ software. Uncropped scans of the blots can be found in Supplementary Fig. 5.

### Real-time PCR

Total RNA was extracted from cultured hfRPE with the RNeasy Mini Kit (Qiagen) and cDNA was prepared with the High Capacity cDNA Reverse Transcription Kit (Life Technologies). Real-time PCR was carried out in a StepOnePlus Real Time PCR System (Life Technologies) using SYBR Select Master Mix (Life Technologies) and the following primer pairs: COL1A1, 5′- cttcacctacagcgtcactg-3′ and 5′-caacgtcgaagccgaattcc-3′; LOX, 5′-catagactgccagtggattg-3′ and 5′-gtgaaattgtgcagcctgag-3′; LOXL1, 5′-tgccagtggatcgacataacc-3′ and 5′-cagtttgttgcagaaacgtagc-3′; LOXL2, 5′-gaggttgcagaatccgattac-3′ and 5′-tgtttaagagcccgctgaag-3′; LOXL3, 5′-ctacattctccaggttgtcatc-3′ and 5′-tagcgttcaaacctcctgttg-3′; LOXL4, 5′-gaagtggcagagtcagatttc-3′ and 5′-ttgttcctgagacgctgttc-3′. GAPDH (5′-ggctggggctcatttgcaggg-3′ and 5′-tgaccttggccaggggtgct-3′) was used as loading control and relative expression values were calculated by the 2^−ΔΔCt^ method.

### Inhibition of lysyl oxidase activity *in vivo*

Newborn (P1) male and female C57BL/6 mice were treated daily with either sterile sodium chloride 0.9% (saline, vehicle control) or BAPN (15 mg kg^−1^ per day, intraperitoneal injection volume 10 μl g^−1^) for 1 month using a 0.5 ml Lo-Dose U-100 insulin syringe equipped with a 28-gauge needle (Becton Dickinson and Company). The dose was chosen based on a previous study^61^. This protocol was reviewed and approved by the Institutional Animal Care and Use Committee. Animals were randomly assigned to the saline- or BAPN-treated groups. After treatment completion, visual function was evaluated (see below), mice were killed and the eyes were enucleated and processed for electron microscopy (see above). Alternatively, haematoxylin and eosin retinal sections were prepared by Excalibur Pathology, Inc. Measurement of retinal and outer nuclear layer thickness was performed at 50 μm intervals and plotted as a function of distance from the optic nerve head.

### Optomotor response analyses

Visual thresholds were measured by evaluating optokinetic tracking in a virtual optokinetic system, as described previously^16^,^62^. Briefly, vertical sine wave gratings (mean white=247.604 candela (cd) m^−2^; mean black=0.260 cd m^−2^) were projected on monitors as a virtual cylinder, which surrounded an unrestrained mouse standing on a platform. An experimenter used a live overhead video image of the arena to view the animal and follow a position between its eyes in real time, using a computer mouse and a crosshair superimposed on the frame. The X–Y positional coordinates of the crosshair centred the hub of the cylinder, enabling its wall to be maintained at a constant ‘distance' from the animal's eyes, thereby fixing the spatial frequency (SF) of the stimulus at the animal's viewing position. When the cylinder was rotated and the animal tracked the rotation with reflexive head and neck movements, it was judged that the animal could distinguish the grating.

Homogeneous grey was presented on the cylinder at the beginning of each testing session. The experimenter waited until the mouse was stationary, at which time grey was replaced with a low SF, high-contrast sine wave grating of the same mean luminance, drifting in one direction. The mouse was assessed for tracking for a few seconds, after which the stimulus restored to grey. The procedure was repeated until unambiguous examples of tracking were observed. The SF of the grating was then increased incrementally, with multiple tests at each SF, until the highest SF that elicited tracking was identified. Thresholds through each eye were measured independently by altering the direction of cylinder rotation, since only temporal-to-nasal motion in the visual field evokes tracking^62^. All experiments were conducted using a cylinder rotation rate of 12 degrees per s, which is a favourable speed to judge optokinetic tracking across a large range of SFs. The experimenter was blind to the treatment condition of the animals.

### ERG tests

After overnight dark adaptation, saline and BAPN treated-mice were anaesthetized with ketamine (100 mg kg^−1^) and xylazine (10 mg kg^−1^) i.p. Eye drops were used to anaesthetize the cornea (1% proparacaine HCl) and the pupils were dilated (1% tropicamide, 2.5% phenylephrine HCl). Mice were placed on a temperature-regulated heating pad throughout each recording session. Retinal function was evaluated by sequentially recording dark- and light-adapted ERG using a Espion E2 System, Diagnosys LLC system. All procedures were performed under dim red light. ERGs were recorded from both eyes using gold wire loops placed on the center of cornea. Reference and ground electrodes were placed subcutaneously in the neck-back region and the base of the tail respectively. ERG was recorded with single-flash delivered in a Ganzfeld dome. For dark-adapted responses (scotopic ERG), stimulus flash intensity varied from 0.01 to 10 cd s m^−2^, with inter-stimulus intervals of 10 to 60 s. Light-adapted ERG responses (photopic ERG) were obtained with flash intensities from 3 to 100 cd s m^−2^, and responses elicited at each intensity were averaged 20 times. Flicker response was elicited on light-adapted mice with a 10-Hz light stimuli (4 ms, 100 cd s m^−2^) on a background of 30 cd s m^−2^. The experimenter was blind to the treatment condition of the animals.

### Generation of polyacrylamide hydrogels

Preparation of polyacrylamide gels was adapted from a previously described protocol^63^. Briefly, the Bio-Rad western blotting system was used to prepare 1 mm-thick gels containing either 8, 15 or 40% acrylamide and 0.264, 1.2 or 3% bis-acrylamide, respectively. Gels were polymerized by the addition of 0.05% APS and 0.1% TEMED. Such gels have been reported to have an approximate modulus of elasticity (E) of 20, 144 and 740 kPa (refs 63, 64, 65). Circular gels were excised using a 1-cm diameter punch and placed in 12-well plates with the aid of forceps. Gels were washed 5 times with double distilled water and left 2–3 days in double distilled water at 4 °C. Gels were placed in the upper chamber of Transwell inserts and rinsed with PBS before surface activation with sulfosuccinimidyl-6-(4′-azido-2′-nitrophenylamino) hexanoate (sulfo-SANPAH, Pierce). Sulfo-SANPAH stock solution (50 mg ml^−1^ in DMSO) was diluted in double distilled sterile water (1 mg ml^−1^) and 250 μl were added to the Transwell upper chamber on top of the gels. Transwells were placed ∼2–3 cm below a UV lamp (254 nm wavelength) for 5 min and gels were rinsed with PBS before repeating the procedure. After washing twice with PBS, 50 μg ml^−1^ rat tail collagen I (Thermo Fisher Scientific) in PBS was added to both chambers of the Transwell inserts and incubated for 3 h at room temperature. Gel-containing Transwells were washed five times with PBS and incubated 30 min at 37 °C in RPE medium before seeding hfRPE cells in the upper chamber, on top of the gel. Cells were cultured for 2 weeks before processing.

### Statistical analyses

All data are presented as mean±s.d. The number of biological replicates (*n*) is indicated in each figure legend. Statistical significance was calculated using two-tailed *t*-test, one-way analysis of variance plus Fisher's LSD *post hoc* analysis or two-tailed N-1 Chi-squared test (*P<0.05; **P<0.01; ***P<0.001) as indicated. In time course assays statistical analyses were carried out for each individual time point among different experimental groups. No statistical method was used to predetermine sample size or to test for normality and variance homogeneity. No samples or animals were excluded from the analyses. RNAseq statistical analyses are detailed in the RNAseq methods section.

### Data availability

All data supporting the findings of this study are available within the article and its Supplementary Information files or upon request. RNAseq data have been deposited in Gene Expression Omnibus (GEO) under series number GSE95835.

## Additional information

**How to cite this article:** Benedicto, I. *et al*. Concerted regulation of retinal pigment epithelium basement membrane and barrier function by angiocrine factors. *Nat. Commun.* **8,** 15374 doi: 10.1038/ncomms15374 (2017).

**Publisher's note:** Springer Nature remains neutral with regard to jurisdictional claims in published maps and institutional affiliations.

## Supplementary Material

Supplementary Information Supplementary Figures

Supplementary Data RNAseq analyses of P5 and P30 mouse choroid ECs. (a) Unfiltered gene expression levels (FPKM) of native P5 and P30 choroid ECs (3 replicates per condition). Values for each replicate and the average and standard deviation (SD) between replicates are shown. (b) Genes differentially expressed (Benjamini-Hochberg corrected P< 0.01) in P5 and P30 choroid ECs (log2 fold change) (see Materials and Methods for filtering information). Highlighted in green, P5>P30; highlighted in blue, P30>P5. (c, d) Gene ontology (GO) analyses using DAVID software of genes enriched in P5 (c) and P30 (d) choroid ECs. Only the categories with Benjamini corrected P<0.01 are shown. (e, f) Genes significantly enriched in P30 choroid ECs that fall into the GO biological process category 'biological adhesion' (e) and the cellular component category 'extracellular matrix' (f).
