# Supplementary material for: Concerted regulation of retinal pigment epithelium basement membrane and barrier function by angiocrine factors
Source: Nat Commun. 2017 May 19;8:15374. doi: 10.1038/ncomms15374 (PMC5454459; doi:10.1038/ncomms15374)
Supplement: Supplementary Information — Supplementary Figures [file ncomms15374-s1.pdf]

Supplementary Figure 1

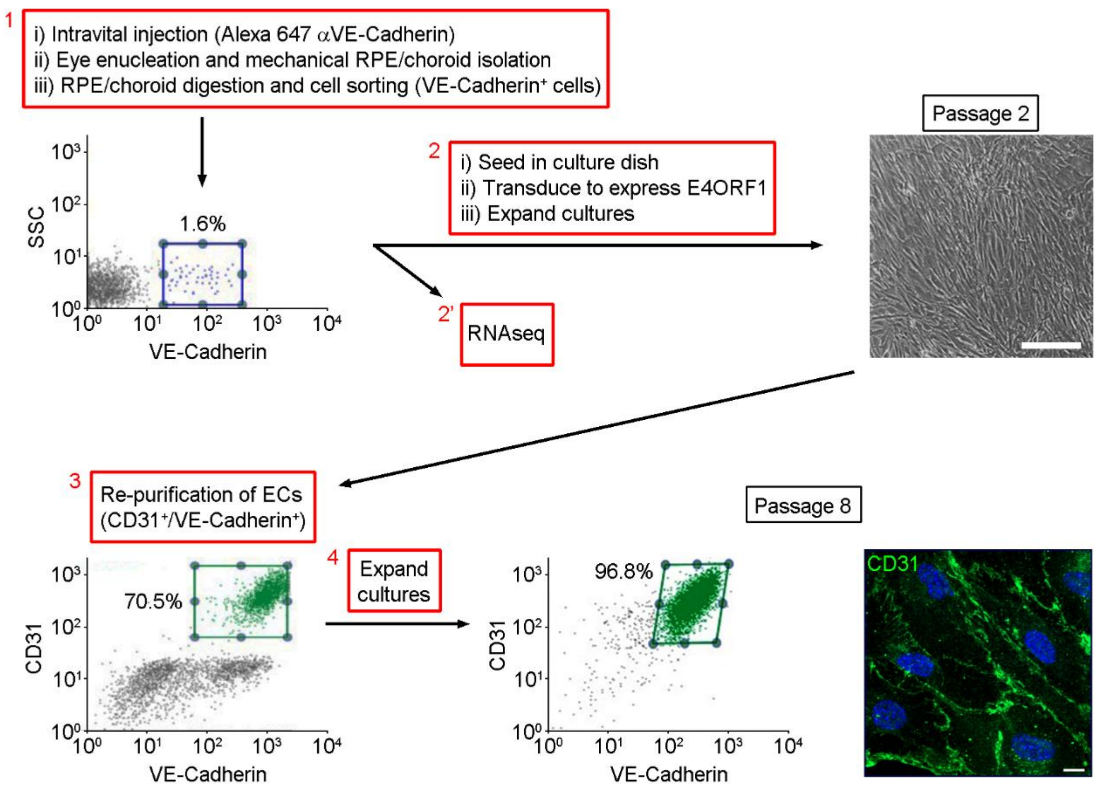

Supplementary Figure 1. Outline of mouse choroid EC isolation, purification and evaluation of purity. Isolated VE-Cadherin$^+$ cells were either used for RNAseq analysis immediately after sorting or seeded to establish cell cultures. Cultured cells were infected with lentivirus encoding the adenoviral protein E4ORF1 and expanded. After 2 passages (bar, 200 µm), ECs were re-purified by cell sorting (CD31$^+$/VE-Cadherin$^+$) and further expanded. After 8 passages, >95% cells were CD31$^+$/VE-Cadherin$^+$ as determined by flow cytometry. A representative immunofluorescence assay shows the presence of CD31 at the plasma membrane of cultured choroid ECs (maximum intensity projection). Bar, 10 µm.

Supplementary Figure 2

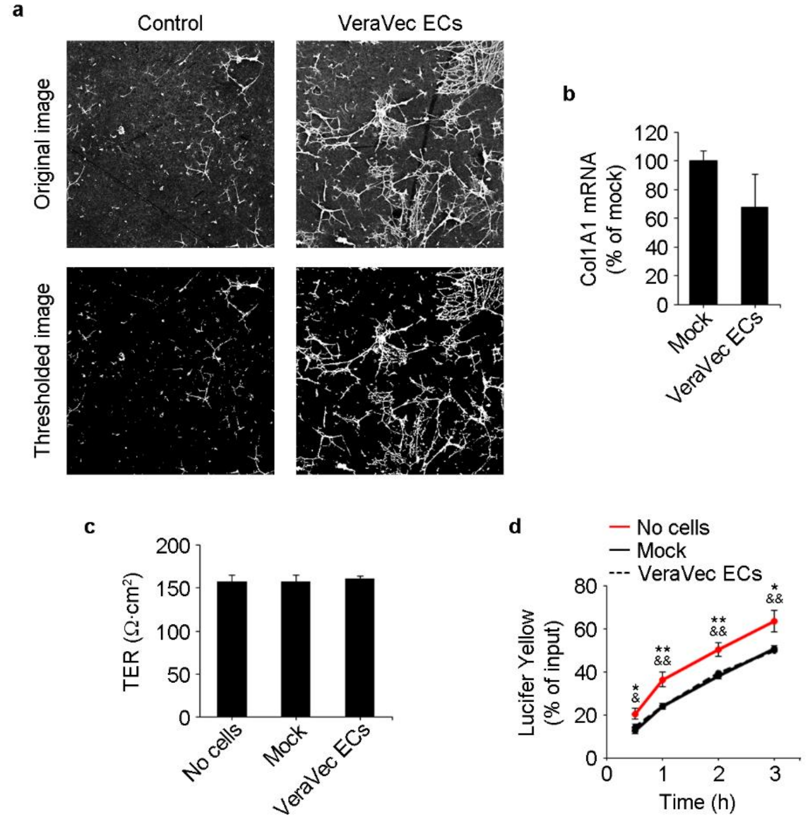

Supplementary Figure 2. EC-induced changes in the ECM beneath hfRPE do not alter *per se* the electrical resistance and permeability of decellularized transwell inserts. (a) Examples of original SEM images of decellularized transwell inserts and the ImageJ-thresholded counterparts. After automatic thresholding, only the pixels corresponding to thick structures (white) were considered for quantification. Note that the original images are the same as the ones shown in Fig. 3d, g. (b) Real time PCR assays showing that EC-conditioned medium does not significantly alter COL1A1 mRNA levels in hfRPE (n=3, t-test). (c, d) Electrical resistance (c) (n=3, ANOVA) and Lucifer Yellow permeability (d) (n=3, ANOVA) across decellularized transwells. Before decellularization, hfRPE cells were cultured in the presence of mock or VeraVec EC-conditioned media for 2 weeks as depicted in Fig. 2b. Bare inserts (no cells) were used as control. In (d), note that permeability across decellularized inserts from hfRPE cultures treated with mock or EC-conditioned media (continuous and dashed black lines) has a lag compared to control inserts (no cells, red line), but after 1 hour the slopes are identical. * and **, no cells vs. mock; & and &&, no cells vs VeraVec ECs. Data are presented as mean ± s.d.

Supplementary Figure 3

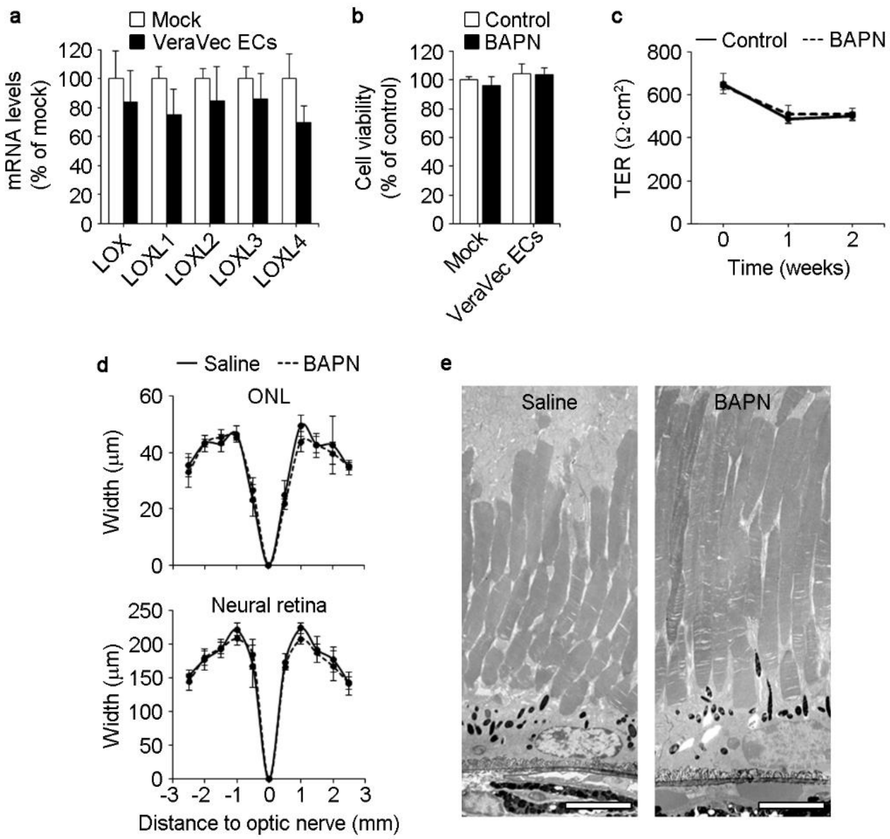

Supplementary Figure 3. Role of lysyl oxidases and effect of BAPN treatment on RPE TER and retinal structure. (a) EC-conditioned medium does not significantly alter LOX, LOXL1-4 mRNA levels in hfRPE. hfRPE cells were cultured in the presence of mock or VeraVec EC-conditioned media for 2 weeks as depicted in Fig. 2b, and hfRPE expression of LOX, LOXL1-4 was assessed by real time PCR (n=3, t-test). (b) BAPN treatment of hfRPE cultured for 2 weeks in the presence of mock or VeraVec EC-conditioned media does not affect cellular viability as measured by Alamar Blue assays (n=3, ANOVA). Data are presented as percentage of mock conditioned media without BAPN. (c) BAPN treatment does not alter TER of fully polarized hfRPE that had been cultured for 2 months before treatment (n=3, t-test). (d) Mice were injected daily with saline or 15 mg kg^{-1} BAPN during the first month after birth. Retinal sections showed no changes in the width of the outer nuclear layer (ONL) or the whole neural retina (n=3, t-test). (e) Transmission electron microscopy showed normal photoreceptor outer segments after BAPN treatment (n=4). Bars, 5 µm. Data are presented as mean ± s.d.

Supplementary Figure 4

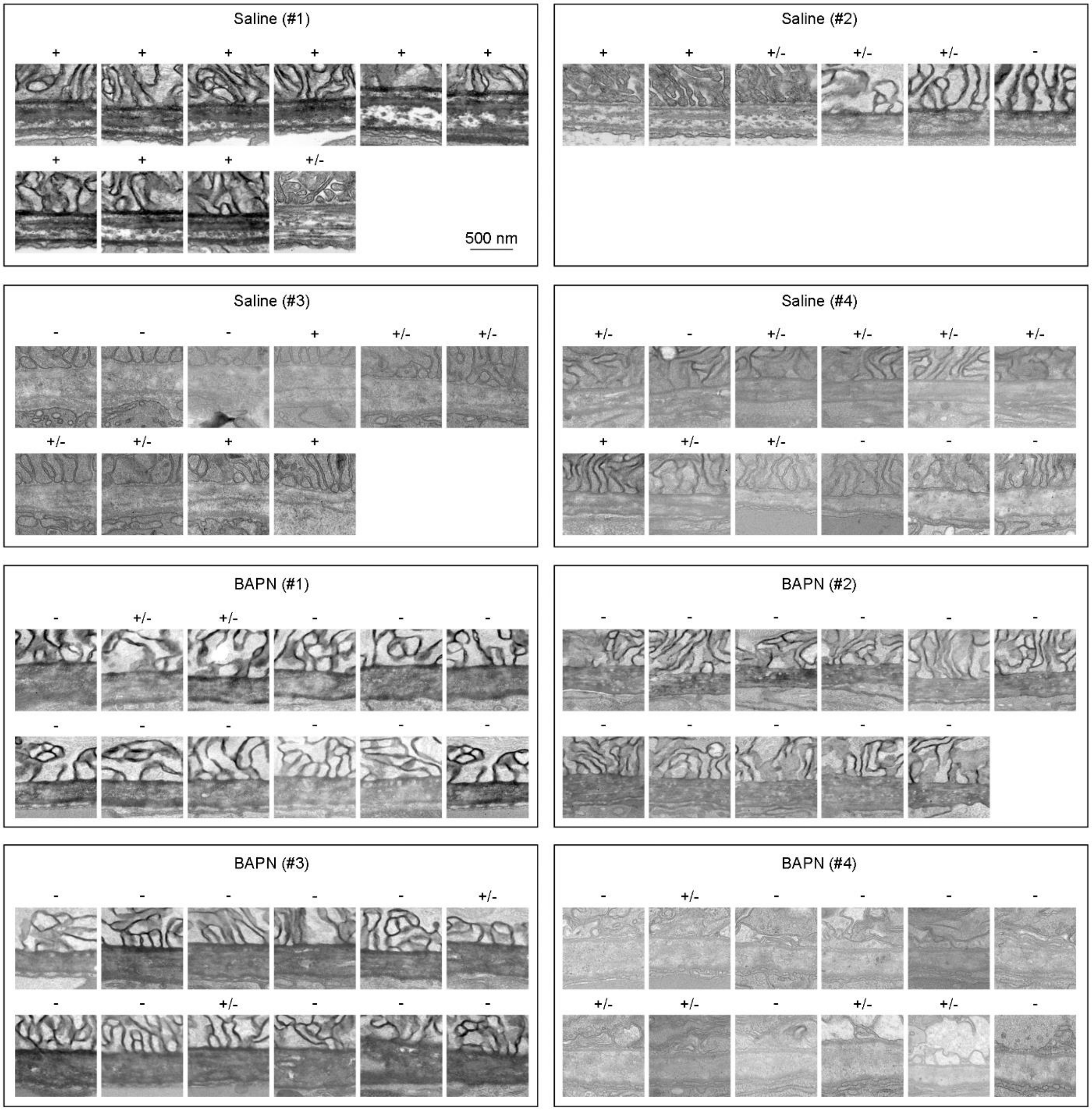

Supplementary Figure 4. BAPN treatment *in vivo* during terminal retinal differentiation induces BM defects. Mice were injected daily with saline or 15 mg kg^{-1} BAPN during the first month after birth. Transmission electron microscopy images show 1 µm-wide BM sections from saline and BAPN-treated P30 animals (4 animals per condition). All images are shown in the same orientation: top, RPE basal membrane; bottom, choriocapillaris. Bar, 500 nm. To quantify BAPN-induced BM alterations, the images were scored as follows: +, evident 5-layered structure; +/-, partial 5-layered structure (discontinuous or faint layers); -, no detectable 5-layered structure. The assigned scores are shown on top of each image.

Supplementary Figure 5

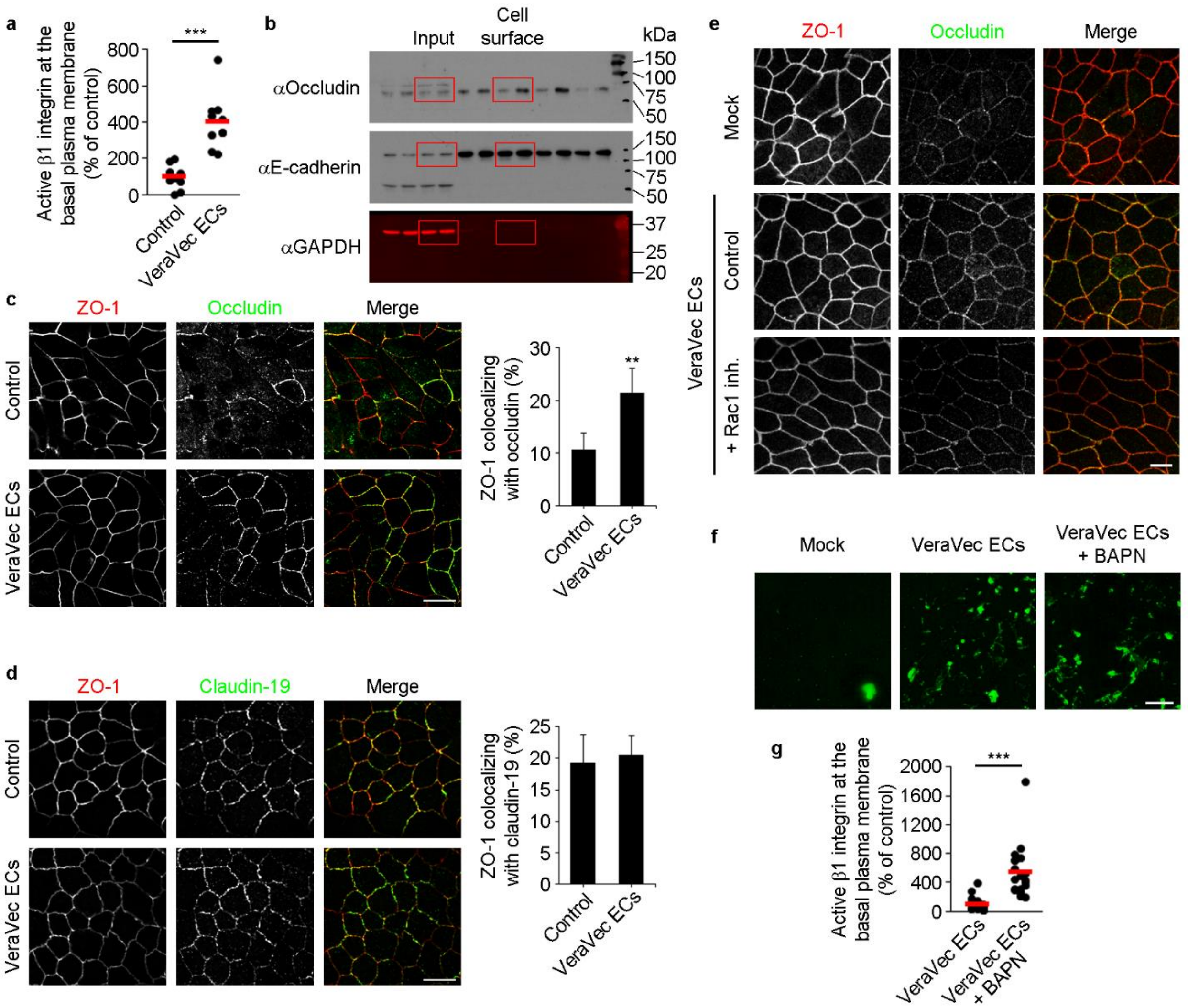

Supplementary Figure 5. Mechanisms involved in EC-mediated RPE TJ regulation. (a) EC-secreted factors increase the levels of active β1 integrin in hfRPE basal plasma membrane. Levels of active β1 integrin (green) and the TJ protein ZO-1 (red) in hfRPE were assessed by immunofluorescence as in Fig. 5a. After quantification, values obtained from all z-stacks were aligned in such a way that all stacks had the xy plane with maximum red (ZO-1) pixel number aligned in the z axis. After calculating the average number of pixels of both channels for each xy plane, we identified the xy plane with maximum average basal active β1 integrin signal. Next, we counted the number of z steps (0.27 µm each) between both maximums and identified the corresponding xy plane of each z-stack. Active β1 integrin values from that xy planes were represented as a percentage of the average control values (10 z-stacks from 2 biological replicates, t-test). (b) Original western blots presented in Fig. 5g (red squares). (c, d) EC-

conditioned media enhances occludin, but not claudin-19, localization at hfRPE TJs. Immunofluorescence assays and confocal analyses of occludin (c) and claudin-19 (d) (green) localization at ZO-1-positive (red) hfRPE TJs after 2 weeks in the absence or presence of VeraVec EC-conditioned media (n=5, t-test). Bar, 20 µm. (e) Rac1 inhibition impairs EC-mediated occludin localization at hfRPE TJs (see quantification in Fig. 5k). Bar, 10 µm. (f) Inhibiton of lysyl oxidase acitivity does not impair EC-mediated accumulation of collagen I in hfRPE basement membrane (see quantification in Fig. 5n). Bar, 10 µm. (g) Inhibiton of lysyl oxidase acitivity further increases the levels of active β1 integrin at hfRPE basal plasma membrane induced by EC-conditioned medium after two weeks in culture. Quantification was carried out as in (a) (20 z-stacks from 2 biological replicates, t-test). Data are presented as mean ± s.d.
